# Adaptive designs for phase II cross-over dose-finding trials using Bayesian model averaging

**DOI:** 10.1186/1745-6215-16-S2-P148

**Published:** 2015-11-16

**Authors:** Sarah Simpson, Lisa Hampson, Thomas Jaki, Byron Jones

**Affiliations:** 1 Lancaster University, Lancaster, UK; 2 Novartis, Basel, Switzerland

## 

Finding the right dose of a novel treatment is one of the most important tasks in early drug development. However, there is often uncertainty about the form of the relationship between dose and patient response at the time that the dose-finding trials are designed. In this presentation we develop Bayesian adaptive designs for Phase II cross-over trials conducted to estimate the minimum effective dose (MED), that is, the dose corresponding to the minimum clinically relevant effect over placebo.

We propose designs which stipulate that patients enter the trial in cohorts, and each cohort receives placebo and three active doses from a total of K active doses available for use. After each cohort of patients has been treated using a Williams square design an interim analysis is performed to identify which doses the next cohort of patients should receive. Bayesian model averaging is used to account for model uncertainty. Prior distributions will be based on pseudo data collected from experts by eliciting quantiles of the response distribution on placebo and a number of active doses to be used in the trial. A number of plausible models for the dose-response relationship are identified before the trial begins and accumulating evidence on the shape of this relationship is summarised by posterior model probabilities. Doses for the next cohort of patients are chosen adaptively to minimise the weighted average variance of the log(MED), averaging across all candidate models and weighting according to posterior model probabilities.

